# The impact of socioeconomic factors on the healthcare costs of people living with HIV in Turkey

**DOI:** 10.1186/s12889-020-08469-z

**Published:** 2020-03-20

**Authors:** Hülya Özkan Özdemir, Selma Tosun, Fatma Nur Karaman Kabadurmuş, Durmuş Özdemir

**Affiliations:** 1 Department of Clinical Microbiology and Infectious Diseases, University of Health Sciences, Bozyaka Education and Research Hospital, İzmir, Turkey; 2 Department of Economics, Yaşar University, Üniversite Caddesi No: 37–39, 35040 Bornova, İzmir, Turkey

**Keywords:** HIV/AIDS, PLHIV, Economic and social factors, Healthcare costs, Turkey

## Abstract

**Background:**

This study addresses an important field within HIV research, the impact of socioeconomic factors on the healthcare costs of people living with HIV/AIDS (PLHIV). We aimed to understand how different socioeconomic factors could create diverse healthcare costs for PLHIV in Turkey.

**Methods:**

Data were collected between January 2017 and December 2017. HIV-positive people attending the clinic who had been referred to the national ART programme from January 1992 until December 2017 were surveyed. The questionnaire collected socioeconomic data. The cost data for the same patients was taken from the electronic database Probel Hospital Information Management System (PHIMS) for the same period. The PHIMS data include costs for medication (highly active antiretroviral therapy or HAART), laboratory, pathology, radiology, polyclinic, examination and consultation, hospitalisation, surgery and intervention, blood and blood products, supplies and other costs. Data were analysed using STATA 14.2 to estimate the generalised linear model (GLM).

**Results:**

The findings of our GLM indicate that age, gender, marital and parental status, time since diagnosis, employment, wealth status, illicit drug use and CD4 cell count are the factors significantly related to the healthcare cost of patients. We found that compared with people who have AIDS (CD4 cells < 200 cells/mm^3^), people who have a normal range of CD4 cells (≥ 500 cells/mm^3^) have $1046 less in expenditures on average. Compared to younger people (19–39 years), older people (≥ 55) have $1934 higher expenditures on average. Costs are $644 higher on average for married people and $401 higher on average for people who have children. Healthcare costs are $518 and $651 higher on average for patients who are addicted to drugs and who use psychiatric drug(s), respectively. Compared to people who were recently diagnosed with HIV, people who were diagnosed ≥10 years ago have $743 lower expenditures on average.

**Conclusion:**

Our results suggest that in addition to immunological status, socioeconomic factors play a substantial role in the healthcare costs of PLHIV. The key factors influencing the healthcare costs of PLHIV are also critical for public policy makers, healthcare workers, health ministries and employment community programs.

## Background

Although, the life expectancy of people living with HIV/AIDS (PLHIV) has increased substantially since the introduction of antiretroviral treatment (ART), current HIV data trends show that a large number of people are still infected and living with HIV in Turkey and in the world, [1–7].

Following these developments, the health care cost of PLHIV is rising and the issue has become increasingly important. Due to the high costs, the incumbent Turkish government currently introduced a cost cutting measure such as the foreign country originated HIV infections are no longer supported with free medical care. This measure has been criticized heavily. The health care costs of PLHIV are classified in a number of different groups in the Probel Hospital Information Management System (PHIMS) [8]. PHIMS data include costs for medication (highly active antiretroviral therapy or HAART), laboratory, pathology, radiology, polyclinic, examination and consultation, hospitalisation, surgery and intervention, blood and blood products, supplies and other costs such as nursing and care services. All state hospitals and number of university hospitals in Turkey use this electronic system to store and share administrative, financial and medical information on a daily basis.

Our study intending to help on cost cutting measures to optimize both sides benefits.

Studies analysing the link between the social and economic factors and the prevalence of a disease are limited in the literature [5, 6, 9–11]. Therefore, the objective of our paper addresses an important field within HIV research, the impact of socioeconomic factors on the healthcare costs of PLHIV in Turkey. To investigate the factors that determine healthcare costs of PLHIV, we considered demographic and socioeconomic characteristics (gender, age, education, marital status, parental status, employment, and wealth), risk factors for HIV infection (smoking, alcoholism, drug addiction, psychiatric drug use, sexual orientation: lesbian, gay, bisexual, and transgender (LGBT) or heterosexual), CD4 T-cell count (at the time of diagnosis), and time since diagnosis (the difference between 2017 and the year when HIV infection was first diagnosed).

Often research in this area focusses on one single issue such as the CD4 cell count and cost or age and cost or employment and cost for PLHIV. There has been no study covering all medical and socioeconomic factors affecting these health care costs. This research not only links the whole set of parameters, but also clarifies the impact of each parameter on the healthcare costs for PLHIV. Different socioeconomic factors can create diverse healthcare costs for PLHIV.

ART improves the quality of life for these patients, yet there are serious cost diversities among PLHIV. The main aim of this study was to clarify the key socioeconomic factors that affect healthcare costs for PLHIV. This may help to plan for future expenditure requirements and to suggest strategies for improving the efficiency of HIV treatment programs.

## Methods

This study is carried out at Izmir Bozyaka Education and Training Hospital, Department of Infectious Diseases and Clinical Microbiology (Izmir, Turkey) where we have a cohort of 153 PLHIV. This cohort characteristics are the same as the whole country and represent the Turkish data in gender and all other sociodemographic factors [4]. Following the ethical committee approval, this study used clinical data recorded by patients and healthcare professionals in the outpatient clinic. In order to determine socioeconomic measures, patients were interviewed face-to-face and the interview questions are the same as the earlier study of Özdemir et.al [9].

Cost data was taken from the electronic database Probel Hospital Information Management System (PHIMS) for the fiscal year 2017 [8]. The data include costs for medication (HAART), laboratory, pathology, radiology, polyclinic, examination and consultation, hospitalisation, surgery and intervention, blood and blood products, supplies and other costs such as nursing and care costs.

Table 1 describes the variables used in our analyses.

**Table 1:** Definition of Variables

VARIABLE	DEFINITION
EXPENDITURES	Total annual healthcare expenditures for 2017 (nominal $).
GENDER	Gender of the patient. Dummy variable = 1 if the patient is Female.
AGE	Age of the patient. Three categories: 1 (19–39), 2 (40–54), 3 (> = 55).
WEALTH	Welfare status of the patient (measured by minimum wage, including non-wage income, family support, transfer payments, etc.). Three categories: 1 (lower than minimum wage), 2 (higher than minimum wage), 3 (higher than double minimum wage).
EDUCATION	Education level of the patient. Two categories: 1 (Middle school or less), 2 (High school and university).
EMPLOYMENT	Employment status. Dummy variable = 1 if the patient is employed, 0 otherwise.
MARITAL STATUS	Marital status. Dummy variable = 1 if the patient is married.
CHILD	Parental status. Dummy variable = 1 if the patient has at least 1 child.
SMOKE	Smoking status. Dummy variable = 1 if the patient is a smoker.
ALCOHOL	Alcohol use. Dummy variable = 1 if the patient drinks alcohol.
DRUG ADDICTION	Drug addiction status. Dummy variable = 1 if the patient is addicted to drugs.
PSYCH DRUG	Psychiatric drug use. Dummy variable = 1 if the patient is taking psychiatric drugs.
SEX ORIENTATION	Sex orientation. Dummy variable = 1 if patient is heterosexual, 0 if identifies as LGBT.
CD4	CD4 T-cell count. Three categories: 1 (< 200 cells/mm3), 2 (200–499 cells/mm3), 3 (≥ 500 cells/mm3).
TIMEDIAG	Duration of treatment. Dummy variable = 1 if the patient was diagnosed ≥10 years ago, 0 otherwise.

Table 2 shows a comparison of total healthcare costs by patient subgroups. Patients with low CD4 T-cell counts and who are female, older, married and have children, who smoke and drink heavily, who are addicted to drugs, who have low-income and education are associated with higher healthcare costs. More specifically, the average healthcare cost is highest ($5422.99) for patients with CD4 cell counts of less than 200 cells/mm^3^. The average cost decreases to $4170.64 as CD4 cell count increases to 200–499 cells/ mm^3^ and is lowest ($3964.49) for patients with CD4 cell count ≥500 cells/ mm^3^. Female patients have a mean healthcare cost of $4689.55 as compared to $4150.25 for males. For patients who are 55 or older, average annual healthcare cost is $5041.74, whereas it is $4820.45 for the 40–54 age group and $3850 for the 19–39 age group. The average healthcare cost is also higher for married people ($4985.74) compared to singles ($3901.95). Looking at healthcare costs of patients with respect to wealth groups, we see that patients in the lowest income group (< Min wage) have the highest costs on average ($4558.78). Other wealth groups with incomes ≥ Min wage and ≥ 2x Min wage have average costs of $4174.21 and $4078.55, respectively. In addition, patients who have children have higher average costs ($4987.97) than patients with no children ($3788.93). The average healthcare cost is higher for smokers ($4383.91) compared to those who don’t smoke ($3985.82). Alcohol users also have a higher average healthcare cost ($4383.90) than non-users ($4186.51). Education level of patients are divided into two sub-groups. Namely, ‘Middle school or less’ and ‘High school and university’. The average healthcare cost is higher for the former group ($4707.58) compared to the latter one ($3918.26). Patients who are employed have higher healthcare cost ($4306.74) compared to employed patients ($4083.07). Patients who identify themselves as LGBT (lesbian, gay, bisexual and transgender), have higher ($4503.50) average healthcare cost than heterosexual patients ($3982.73). Lastly, the average healthcare cost for patients who were diagnosed ≥10 years ago is higher ($4291.69) than the cost for patients who were diagnosed more recently ($4220.34).

**Table 2:** Baseline characteristics of participants (*n* = 153, cost in $)

PATIENT TYPE	Mean Cost ($)	Std. Dev.	Freq.
GENDER			
Male	4150.25	2248.05	131
Female	4689.55	1331.37	22
AGE			
19–39 years	3850.03	2177.93	98
40–54 years	4820.45	2081.52	35
≥ 55 years	5041.74	1666.04	20
WEALTH			
< Min wage	4558.78	1826.99	35
≥ Min wage	4174.21	2252.34	63
≥ 2x Min wage	4078.55	2221.23	55
CD4			
< 200 cells/mm^3^	5422.99	1883.97	19
200–499 cells/ mm^3^	4170.64	2112.31	61
≥ 500 cells/ mm^3^	3964.49	2159.23	73
SMOKING			
No	3985.82	2085.15	60
Yes	4383.91	2180.40	93
ALCOHOL USE			
No	4186.51	1970.35	121
Yes	4383.90	2740.54	32
DRUG ADDICTION			
No	4167.50	2066.87	123
Yes	4475.01	2465.13	30
PSYCHIATRIC DRUG USE			
No	4144.39	2142.92	135
Yes	4853.35	2119.43	18
EDUCATION			
Middle school or less	4707.58	2144.43	60
High school and university	3918.26	2099.81	93
EMPLOYMENT			
Unemployed	4083.07	2023.49	54
Employed	4306.74	2215.28	99
MARITAL STATUS			
Single	3901.95	2071.78	107
Married	4985.74	2144.35	46
SEXUAL ORIENTATION			
LGBT	4503.50	1877.71	72
Heterosexual	3982.73	2342.59	81
HAVE CHILDREN			
No	3788.93	2151.65	97
Yes	4987.97	1926.85	56
TIME SINCE DIAGNOSIS			
< 10 years	4220.34	2184.93	137
≥ 10 years	4291.69	1832.35	16

Abbreviations: *Min wage* minimum wage, *CD4* CD4 T-cell count, *LGBT* lesbian, *gay* bisexual and transgender, *Std. Dev* standard deviation, *Freq*. frequency

**Fig. 1:**
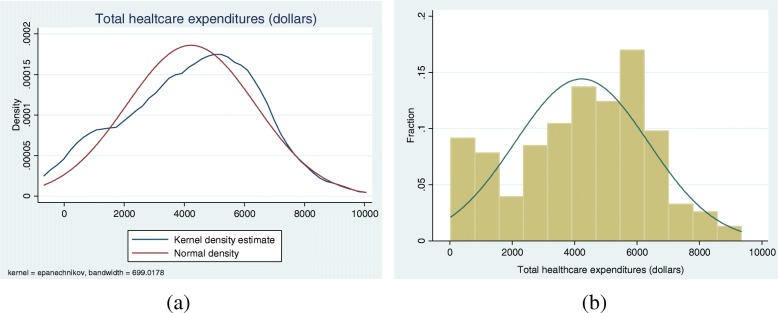
Distribution of total healthcare costs

Our dependent variable measures total annual health care expenditures for 2017, measured in nominal US dollars. Fig. 1 shows that the distribution is left-skewed, and not symmetric.^1^

Although our data is not highly skewed, we avoided using ordinary least squares (OLS). Instead, we used generalised linear models (GLM) following Deb and Norton [12]. to relax the OLS assumptions of homoscedasticity and normality. First introduced by Nelder and Wedderburn [13], the GLM generalises the linear model and specifies the relationship between the observed response variable and some number of covariates. The GLM has two components: a link function, which links the expected value of the outcome to the linear predictor comprising the regression coefficients; and a variance function, which relates the variance as a function of the mean and is specified using the name of a particular member distribution of the exponential family [14].

To describe the form of the GLM, we first start with the classical linear model which can be summarized as:

$$ E(Y)=\mu\ where\ \mu = x\beta .\kern0.75em (1) $$

The components of Y are independent normal variables with constant variance σ^2^. To generalize eq. (1), a three-part specification can be used:

(1) The random part: The components of Y have independent normal distributions with *E*(*Y*) = *μ* and constant variance σ^2^.

(2) The systematic component: covariates x_1_, x_1_, …, x_p_ produce a linear predictor η given by

$$ \eta =\sum \limits_{j=1}^p{x}_{ij}{\beta}_{j.} $$

(3) The link between the random and the systematic components:

$$ \mu =\eta . $$

If we write *η*_*i*_ = *g*(*μ*_*i*_), then *g(.)* is the link function and Y~F (distribution, eg., normal, binomial, Poisson, etc.). In this formulation, classical linear models have a Gaussian (Normal) distribution in the random part and the identity function for the link component [15]. To estimate the GLM model, we used Newton–Raphson (maximum likelihood) optimization. All analyses were performed in STATA 14.2.

As explained in the preceding paragraphs, constructing the GLM involves selecting a variance function and an appropriate link function. We used common specification tests to make these choices. We first used the Box-Cox test [16] to determine whether our dependent variables needed any transformation in order to have a symmetric distribution. We did the Box-Cox test in two ways, with and without controlling for covariates. The test results showed that the linear, log, and multiplicative inverse specifications are strongly rejected. Combining these with what Fig. 1 reveals about our cost data, we concluded that our cost variable does not need any transformation.

Next, we proceeded with the choice of the distribution family. We used a modified Park test [17–19] after running a GLM with gamma family and log link. The procedure involves computing the mean (expected value) and variance (squared error) for each observation and then predicting the squared error as a function of the expected value. The coefficient on the expected value indicates distribution. If the value is close to 0.0, it indicates a Gaussian family in which the variance is constant. If the value is close to 1.0, it indicates a Poisson-like family in which variance is equal (or proportional) to the mean. A value of 2.0 or 3.0 indicates a Gamma (variance is proportional to the mean squared) and inverse-Gaussian (variance is proportional to the cube of the mean) distribution, respectively [19]. Our estimated coefficient was equal to 0.87, therefore our sample follows Poisson distribution.

As Glick et al. [19] point out, misspecification of family may result in efficiency losses, but it does not affect consistency as long as the link function is correctly specified. The power link has an important role in assessing the fit of models. It allows one to generate a wide variety of links. For example, power = 1, power = 0, and power = − 1 corresponds to the identity link, the log link and the reciprocal link, respectively [14]. To find the optimal link, we compared deviance and log-likelihood values and AIC and BIC statistics. Moreover, we implemented Pregibon’s link test [20] which evaluates the linearity of the response on the scale of estimation. The test assesses whether the coefficient on the squared term is significantly different from zero. We evaluated the power links in increments of 0.1 between − 2 and 2 to choose the most appropriate link [14]. These statistics are presented in Table 3 for a selected set of power links. We do not report the statistics for all power links in order to ease presentation. Log-likelihood, deviance, AIC and BIC statistics all suggest to choose power link (1.4). However, as Glick [21] argues, the first four statistics are not sufficient alone to identify the appropriate link function. Thus, we proceed and choose the optimal link as (1.3), in line with the results of Pregibon’s link test. Moreover, there is not much variation in magnitudes of the four statistics for power link (1.3) and power link (1.4).

**Table 3:** Optimal Link Selection

	POWER (−1)	POWER (−0.5)	POWER (0)	POWER (0.5)	POWER (1)	POWER (1.1)	POWER (1.2)	POWER (1.3)	POWER (1.4)
AIC	1123	1114	1104	1092	1077	1073	1070	1066	1061
BIC	169,561	168,261	166,695	164,832	162,532	161,992	161,414	160,793	160,137
Log-likelihood	−85,879	−85,229	−84,446	−83,514	−82,364	−82,094	−81,805	−81,495	−81,167
Deviance	170,240	168,940	167,374	165,511	163,211	162,671	162,093	161,472	160,816
Pregibon’s test, *P*-value for squared term	0.178	0.305	0.546	0.323	0.421	0.475	0.626	0.906	0.754

Abbreviations: *AIC* Akaike information criterion, *BIC* Bayesian information criterion. Initial values were not feasible to fit GLM for power ≥ 1.5. Power links greater than 1 are usually appropriate for data having a response with a sharp increase of values (Hardin and Hilbe, 2018)

## Results

Table 4 reports our GLM results for Poisson distribution and power link (1.3), which is the most appropriate model based on the aforementioned criteria. Incremental effects are reported in column 2 of Table 4. As a robustness check, we have also presented results for identity and log links in Appendix Table 5.

**Table 4:** Generalised linear model (GLM) results

	(1)^a^			(2)
VARIABLES/LINK^b^	POWER (1.3)	%95 Confidence Interval		Incremental Effects
CD4 (200–499 cells/mm^3^)	−14,556.84***	−15,199.1	−13,914.6	− 898.1***
	− 327.678			−19.83
CD4 (≥ 500 cells/mm^3^)	−16,868.45***	−17,503.1	− 16,233.8	−1046***
	−323.80			−19.59
Wealth (≥ min wage)	−21,868.29***	−22,411.3	−21,325.3	− 1365***
	− 277.05			−17.08
Wealth (≥ 2x min wage)	−20,391.69***	−20,940.9	−19,842.5	− 1268***
	− 280.21			−17.24
Employment (employed)	22,637.573***	22,206.9	23,068.25	1482***
	−219.74			−14.93
Education (high school & university)	911.83***	512.04	1311.62	58.24***
	−203.98			−13.04
Alcohol (yes)	1439.45***	996.98	1881.92	91.65***
	− 225.75			−14.34
Smoke (yes)	7150.11***	6780.94	7519.27	458.0***
	−188.35			−12.12
Psychiatric drug (yes)	10,415.17***	9844.42	10,985.91	651.3***
	−291.20			−17.87
Drug addiction (yes)	8225.30***	7755.71	8694.90	518.1***
	− 239.59			−14.91
Sexual orientation (heterosexual)	− 575.12***	− 983.17	−167.06	−36.69***
	−208.20			−13.28
Age (40–54)	14,638.10***	14,169.67	15,106.53	932.0***
	− 238.10			−15
Age (≥ 55)	31,475.19***	30,741.65	32,208.73	1934***
	−374.26			−22.18
Gender (female)	13,398.90***	12,782.68	14,015.13	835.1***
	− 314.41			−19.18
Marriage status (married)	10,222.66***	9720.721	10,724.59	643.6***
	− 256.09			−15.92
Child (yes)	6337.71***	5809.123	6866.289	401.4***
	− 269.69			−16.97
Time diagnosed ^c^ (≥ 10 years)	−11,356.30***	−11,967.4	− 10,745.2	− 742.19***
	−311.79			73.37
Constant	46,693.04***	45,804.71	47,581.38	4225***
	− 453.24			−5.254
Observations	153			153
AIC	1066			
BIC	160,793			
Log-likelihood	−81,495			
Deviance	161,472			

Abbreviations: *Min wage* minimum wage, *CD4* CD4 T-cell count, *AIC* Akaike information criterion, *BIC* Bayesian information criterion

^a^ *** *p* < 0.01

^b^ Parentheses show the categories of the independent variables that are included in the estimations. Reference categories are omitted

^c^ Margins command in STATA is unable to produce incremental effects for the variable *Timediagnosed*. We therefore calculated it by hand following Williams [22]

Incremental effects (marginal effects for categorical independent variables) show how the probability of outcome changes given a unit change in the value of the categorical variable. We calculate these as average marginal effects (AMEs). Thus, the marginal effect is first calculated for each individual with their observed levels of covariates. These values are then averaged across all individuals. For the interpretation of AMEs for Poisson regression GLM, we followed Hardin and Hilbe [14].

The incremental effects reported in Table 4 reveal that age, gender, employment status, wealth and CD4 count are the most important factors in determining the healthcare costs of PLHIV. This shows that the individual characteristics of patients are significantly related to costs.

As expected, a better health status of the patient, measured by CD4 cell count, decreases healthcare costs. We found that compared with people who have AIDS (CD4 cells < 200 cells/mm^3^), people who have a normal range of CD4 cells (≥ 500 cells/mm^3^) have $1046 less in expenditures on average (Table 4, column 2). This result is in agreement with the findings of Dube et al. [23] who conducted a systematic review of predictors of HIV infection and argued that early diagnosis and earlier use of therapies improve the effectiveness of treatment and thus reduce healthcare costs.

Demographic variables include age, gender, marital and parental status. Age has the largest effect on costs. Compared to younger people (19–39 years), older people (≥ 55 years) have $1934 higher expenditures on average. This result matches those observed in earlier studies. As older people are more susceptible to infections [24] and have an immunologic response to therapy [25, 26], they incur higher medication costs [27].

We also found that health care costs are $835 higher on average for females. This finding is consistent with previous works demonstrating that women are more prone to the risks of HIV infection compared to men [28–30]. Prior research also argues that women have better health maintenance compared to men and have greater utilisation of healthcare services [31–34].

Marital and parental status are also associated with higher healthcare costs. Costs are $644 higher on average for married people and $401 higher on average for people who have children. Earlier studies have shown that the odds of HIV infection are higher for married women [35], as they cannot refuse sex and ask for condom use in marriage [36]. In addition, it is possible to hypothesise that people with children have a higher risk of acquiring infectious diseases.

Socioeconomic status variables include education, wealth and employment status. Care costs are $1482 higher on average for employed and $58 higher on average for people that are more educated. Since both education and employment indicate a higher socioeconomic status, these people could be better informed and have more access to care services, which would explain higher care costs [37]. Wealth represents the welfare status of an individual (measured by minimum wage, including non-wage income, family support, transfer payments, etc.) and is divided into 3 categories: lower than minimum wage, higher than minimum wage and higher than double minimum wage. Healthcare costs are significantly lower for wealthier people. An implication of this is that people with lower income levels live in communities with lower socioeconomic status and have a higher risk of acquiring infections [38, 39].

Risk factors include sexual orientation, smoking, alcohol use, drug addiction and psychiatric drug use. Of all these, psychiatric drug use and drug addiction are the two major factors that increase healthcare costs. Healthcare costs are $518 and $651 higher on average for patients who are addicted to drugs and who use psychiatric drug(s), respectively. Earlier studies have also demonstrated that drug use is significantly and positively related to HIV sexual risk behaviour [40], and injection drug use is a mode of HIV transmission in many regions including China, the USA, and Russia [41]. In addition, substance users have multiple comorbidities that complicate HIV treatment and prevention [42] and are particularly vulnerable to suboptimal combination antiretroviral therapy (cART) adherence [43].

Smoking and alcohol use are also common risk factors among PLHIV, and they contribute to increased incidence of non-AIDS-related morbidity and mortality [44, 45]. In our sample, smoking status seemed to have a greater impact on costs compared to alcohol use.

Sexual orientation poses another risk factor. In our sample, we found that people who identify themselves as heterosexual have slightly lower healthcare expenditures compared to LGBT people. Homosexual people could be better informed about access to healthcare and thus have higher costs [34]. At the same time, they have riskier sexual behaviour, which increases the prevalence of HIV and sexually transmitted infections (STIs) and thus overall care costs [46–48].

Finally, we consider the duration of treatment. Compared to people who were recently diagnosed with HIV, people who were diagnosed ≥10 years ago have $743 lower expenditures on average. This may be due to the improvements in CD4 count with the number of years from diagnosis [31].

## Discussion

This study provides valuable data about the link between socioeconomic factors and the healthcare costs of PLHIV. In addition, we have shown that the findings of our GLM indicate that these socioeconomic parameters and CD4 cell count are the factors significantly related to the healthcare costs of patients. A number of studies show that HAART reduces the average number of annual hospitalisations, and hence results in considerable cost savings [49–51]. But the literature lacks the information regarding PLHIV already on HAART and how other factors, such as socioeconomic, are affecting the healthcare costs of PLHIV. The findings of this study strongly suggest that the healthcare costs of PLHIV is associated with socioeconomic parameters such as age, gender, marital and parental status, time since diagnosis, employment, wealth status and illicit drug use.

Our results confirm the literature [23] on the link between the better health status of the patient decreases healthcare costs. Age has the largest effect on costs in this study. Compared to younger people, older people have higher expenditures. As older people are more susceptible to infections and have an immunologic response to therapy, they incur higher medication costs. Following sources [24–27] also confirm these results. Female healthcare costs are higher as opposed to male health care costs. Prior research also argues that women have better health maintenance compared to men and have greater utilisation of healthcare services [28–34]. Marriage increases the costs; the odds of HIV infection are higher for married women as they cannot refuse sex and ask for condom use in marriage. Earlier studies have supported these results [35, 36]. Both education and employment indicate a higher socioeconomic status, educated and employed people could be better informed and have more access to care services, which would explain higher care costs [37]. Healthcare costs are significantly lower for wealthier people. An implication of this is that people with lower income levels live in communities with lower socioeconomic status and have a higher risk of acquiring infections [38, 39];. Drug use is significantly and positively related to HIV sexual risk behaviour and causes higher costs. In addition, substance users have multiple comorbidities that complicate HIV treatment and prevention and are particularly vulnerable to suboptimal cART adherence. Earlier studies have also supported the link between drug use and HIV costs [40–43]. Smoking and alcohol use contribute to increased incidence of non-AIDS-related morbidity and mortality [44, 45]. In our study, smoking status seemed to have a greater impact on costs compared to alcohol use. We found that people who identify themselves as heterosexual have slightly lower healthcare expenditures compared to LGBT people. Homosexual people have higher costs. They are better informed about access to healthcare, they have riskier sexual behaviour, which increases the prevalence of HIV and sexually transmitted infections (STIs) and thus overall care costs are higher [34, 46–48]. The duration of treatment lowers the expenditures. This may be due to the improvements in CD4 count with the number of years from diagnosis [31].

Together, these results provide important insights into the factors that affect healthcare costs of PLHIV. We have demonstrated that in addition to immunological status of patients, socioeconomic variables are important factors that determine costs of PLHIV.

The policy implications of the key factors influencing the healthcare costs of PLHIV are also critical for public policy makers, healthcare workers, health ministries and employment community programs.

This study is not without its limitations. The clinic and the country were not selected at random but they represent the whole country [48]. and as such this is a convenience sample; therefore, some of the estimates may have been over- or underestimated. The proportion of female PLHIV is low compared to male PLHIV. The total sample of PLHIV in our data is small, and this may cause unobserved discrepancies in our results. Costs may vary in other settings depending on drug costs and administrative policies.

## Conclusion

Our results suggest that in addition to immunological status, socioeconomic factors play a substantial role in the healthcare costs of PLHIV.

As expected, a better health status of the PLHIV, measured by CD4 cell count, decreases healthcare costs but socio-economic factors such as the age, gender, marital and parental status, smoking, alcohol use, time since diagnosis, employment, wealth status, sexual orientation and illicit drug use are also having a crucial role for decreasing the costs of PLHIV.

The key factors influencing the healthcare costs of PLHIV are also critical for public policy makers, healthcare workers, health ministries and employment community programs.

## Data Availability

The dataset used and analyzed during the current study are available from the corresponding author on reasonable request.

## Appendix

**Table 5:** GLM Results for Identity and Log Links

	(1)	(2)
VARIABLES/LINK	IDENTITY	LOG
CD4 (200–499 cells/mm^3^)	− 789.194***	−0.128***
	−19.659	−0.004
CD4 (≥ 500 cells/mm^3^)	− 951.866***	−0.172***
	−19.447	− 0.004
Wealth (≥ min wage)	− 1270.716***	− 0.218***
	−17.79	− 0.004
Wealth (≥ 2x min wage)	− 1218.168***	− 0.219***
	−18.536	− 0.004
Employment (employed)	1349.770***	0.242***
	−15.413	−0.004
Education (high school & university)	−33.103***	−0.042***
	−12.839	−0.003
Alcohol (yes)	166.506***	0.074***
	−14.565	−0.004
Smoke (yes)	435.100***	0.110***
	−12.397	−0.003
Psychiatric drug (yes)	581.622***	0.078***
	−17.829	−0.004
Drug addiction (yes)	498.590***	0.091***
	−14.872	−0.003
Sexual orientation (heterosexual)	106.991***	0.090***
	−13.437	−0.003
Age (40–54)	860.885***	0.174***
	−14.732	−0.003
Age (≥ 55)	1912.262***	0.384***
	−23.162	−0.005
Gender (female)	870.202***	0.200***
	−19.715	−0.004
Marriage status (married)	723.812***	0.199***
	−15.501	−0.003
Child (yes)	343.916***	0.058***
	−16.673	−0.004
Time diagnosed (≥ 10 years)	− 639.422***	−0.095***
	−19.443	−0.004
Constant	3733.697***	8.148***
	−28.266	−0.007
Observations	153	153
AIC	1077	1104
BIC	162,532	166,695
Log-likelihood	−82,364	−84,446
Deviance	163,211	167,374

Abbreviations: *Min wage* minimum wage, *CD4* CD4 T-cell count, *AIC* Akaike information criterion, *BIC* Bayesian information criterion

## Abbreviations

PLHIV: People living with HIV/AIDS
PHIMS: Probel Hospital Information Management System
HAART: Highly Active Antiretroviral Therapy
GLM: Generalised Linear Model
LGBT: Lesbian, Gay, Bisexual, Transgender
OLS: Ordinary Least Squares
cART: Combination Antiretroviral Therapy
STIs: Sexually Transmitted Infections
Min wage: Minimum Wage
CD4: CD4 T-cell count
Std. Dev: Standard deviation
Freq: Frequency
AIC: Akaike Information Criterion
BIC: Bayesian Information Criterion

## Footnotes

1 For the sample, skewness = − 0.16 and kurtosis = 2.4. We have also used Skewness and Kurtosis Test for normality after fitting a linear model. The null hypothesis which states that the data follow a normal distribution is rejected at a 5% significance level.
